# Deciphering the Role of Ferroptosis in the Pathogenesis of Peripheral Artery Disease Myopathy

**DOI:** 10.3390/biology14050537

**Published:** 2025-05-12

**Authors:** Trevor Wilkinson, Emma Fletcher, Andrew Ring, Cassandra Bradley, Evlampia Papoutsi, Dimitrios Miserlis, Robert S. Smith, William T. Bohannon, Iraklis I. Pipinos, Panagiotis Koutakis

**Affiliations:** 1 Department of Biology, Baylor University, Waco, TX 76798, USA; 2 Department of Public Health, University of West Florida, Pensacola, FL 32514, USA; 3 Department of Surgery and Perioperative Care, Dell Medical School, University of Texas, Austin, TX 78712, USA; 4 Department of Surgery, Baylor Scott and White Medical Center, Temple, TX 76508, USA; 5 Department of Surgery, University of Nebraska at Medical Center, Omaha, NE 68198, USA

**Keywords:** ferroptosis, peripheral artery disease, iron metabolism, oxidative stress, lipid peroxidation, autophagy

## Abstract

This study investigates ferroptosis in the context of peripheral artery disease (PAD), a vascular disease characterized by atherosclerosis of the lower extremities. Muscle atrophy and increased oxidative stress are hallmarks of PAD and correlate with worse clinical outcomes. Given ferroptosis’ association with oxidative stress, we explored its role in PAD myopathy by examining gene and protein markers related to metabolic pathways implicated in ferroptosis using both human PAD patients and cultured myotubes. Intermittent claudication (IC) PAD patients, critical limb ischemia (CLI) PAD patients, and non-PAD controls were recruited for this study. Calf muscle biopsies were analyzed for gene expression using qPCR, and protein levels were determined by Western blotting. Cultured myotubes treated with the ferroptosis inducer erastin provided an in vitro comparison. Results demonstrated upregulation of ferroptosis markers such as lipid peroxidation and *PTGS2* gene expression in the muscle of CLI PAD patients compared to controls. Increased expression of ferroptosis-related genes *HMOX1, ACSL4, ELAVL1*, and *Beclin-1* was also observed. Protein analysis showed trends consistent with gene expression in some ferroptosis markers. The increase in ferroptosis markers in CLI PAD patients, particularly in iron metabolism and autophagy pathways, suggests ferroptosis contributes to PAD myopathy.

## Simple Summary

This study investigates the role of ferroptosis in peripheral artery disease-driven myopathy. Ferroptosis is a form of cell death characterized by iron-dependent lipid peroxidation. In this study, we tested leg muscle tissue from peripheral artery disease patients for different ferroptosis markers to determine whether ferroptosis plays a role in this myopathy. General markers of ferroptosis—such as lipid peroxidation—were increased in the muscle tissue of advanced peripheral artery disease patients. Specific markers related to iron metabolism and autophagy were also elevated. Based on these findings, we conclude that ferroptosis is a component of peripheral artery disease myopathy, and that ferroptosis is a promising new therapeutic avenue for peripheral artery disease research.

## 1. Introduction

Peripheral artery disease (PAD) is an atherosclerotic disease characterized by the narrowing of the blood vessels of the lower extremities [1,2]. PAD affects between 5.8% and 10.7% of individuals over the age of forty in the United States, and over 230 million people worldwide [2,3,4]. Previous studies show that PAD causes a myopathy associated with oxidative stress [5,6,7,8,9,10], inflammation [11,12,13,14,15], and abnormal myofiber morphology [16,17,18], which increase as the disease progresses, as well as increased mortality rates [8,19,20,21]. Therefore, understanding the mechanisms that connect oxidative stress to myopathic development in PAD is essential to better understand the disease.

Ferroptosis is a recently defined form of non-apoptotic cell death driven by iron-dependent lipid peroxidation [22]. This process involves the accumulation of lipid peroxides due to alterations in four overlapping metabolic pathways: iron metabolism, antioxidant metabolism, lipid metabolism, and autophagy. Ferroptosis is already implicated in several PAD-related conditions, including sarcopenia, inflammation, ischemia/reperfusion injury (IRI), and atherosclerosis [23,24,25,26,27,28]. To date, only one study has investigated the link between ferroptosis and PAD myopathy. The investigators demonstrated that genes associated with iron metabolism were upregulated in satellite cells from the calf muscle of patients with severe PAD, while glutathione peroxidase 4, a major antioxidant and inhibitor of ferroptosis, was downregulated [29]. This study provides the first evidence that ferroptosis is a component of severe PAD myopathy; however, further investigation is warranted. Therefore, the primary objective of the current study was to investigate whether ferroptosis markers were altered in muscle tissue from patients with lower severity PAD myopathy, i.e., patients with intermittent claudication (IC), and whether ferroptosis progresses as PAD myopathy progresses from IC to critical limb ischemia (CLI) disease. We also compared any alterations noted in patient samples with those from cultured myotubes treated with the ferroptosis inducer erastin.

## 2. Materials and Methods

The data that support the findings of this study are available from the corresponding author upon reasonable request.

### 2.1. Study Population

For this analysis, data from PAD patients originally recruited for the human clinical trial (NCT04089943) were used. Vascular surgeons at Baylor Scott and White Hospital and the University of Texas at Austin Dell Medical School recruited patients with symptomatic infrainguinal PAD and CLI patients displaying arterial insufficiency with gangrene, non-healing ulcers, and/or consistent rest pain. All diagnoses were made following physical and medical history examination, ankle–brachial index (ABI) measurement, and arteriography. Healthy adults with normal blood flow to their extremities were recruited as well. Patients with any musculoskeletal symptoms, neurological symptoms, or acute lower extremity ischemic events secondary to thromboembolic disease or trauma were excluded from this study. This study was approved by the Institutional Review Board of the University of West Florida and was carried out in accordance with relevant guidelines and regulations governing human research. This study complies with the Declaration of Helsinki, and informed consent was obtained from all participants.

### 2.2. Muscle Biopsy

Biopsies were obtained from the anteromedial segment of the gastrocnemius, approximately 10 cm distal to the tibial tuberosity. A fine needle (12 G) was used to obtain a total sample of ~250 mg. Approximately 200 mg was snap-frozen in liquid nitrogen for later analysis.

### 2.3. Myotube Cell Culture

Skeletal muscle-derived cells from healthy adults (Cook Myosite, Pittsburgh, PA, USA, SK-1111) were cultured at 37 °C under standard cell culture conditions (20% O_2_, 5% CO_2_). The culture medium consisted of Ham’s Nutrient Mixture (Cytiva, Logan, UT, USA, SH30025.01), 20% fetal bovine serum (FBS) (Corning, Durham, NC, USA, 35-011-CV), 1% Penicillin–Streptomycin (Pen–Strep) solution (Thermo Fisher, Waltham, MA, USA, 11550-043, 26140-079, 15140-122), and 5 ng/mL Fibroblast Growth Factor-basic (FGF-b) (Sigma-Aldrich, St. Louis, MO, USA, GF003). Cells were plated on T-75 flasks coated with 0.1% gelatin/phosphate-buffered saline (PBS) solution (ATCC, Manassas, VA, USA). The culture medium was replaced every other day with fresh medium; cells were subcultured when cultures reached approximately 80% confluence. Cells were eventually split into tissue-culture-treated 6-well plates (Celltreat, Ayer, MA, USA, 229106), where they were treated with differentiation media that consisted of high glucose Dulbecco’s Modified Eagle Medium (DMEM) (Cytiva, Logan, UT, USA, SH30243.01), 2% Horse Serum (Innovative Research Inc., Novi, MI, USA, IGHSSER500ML), 1% Pen–Strep solution (Thermo Fisher, 11550-043, 26140-079, 15140-122), 0.2% antibiotic/antimycotic solution (Cytiva, Logan, UT, USA, SV30079.01), and 0.005% insulin (Thermo Fisher, MP219390010) for four days. Once the cells had differentiated, they were treated with either 5 µM erastin (erastin myotubes) (TargetMol, Boston, MA, USA, T1765), a ferroptosis promoter [30], or an equivalent volume of FBS for 24 h (FBS myotubes), then harvested for either RNA or protein extraction.

### 2.4. RNA Isolation and qPCR

To determine the relative expression of heme oxygenase 1 (*HMOX1*), transferrin receptor 1 (*TFRC*), ferritin heavy chain (*FTH1*), nuclear receptor co-activator 4 (*NCOA4*), solute carrier family 40 member 1 (*SLC40A1*), glutathione peroxidase 4 (*GPX4*), solute carrier family 7 member 11 (*SLC7A11*), long-chain-fatty-acid—CoA ligase 4 (*ACSL4*), prostaglandin-endoperoxide synthase 2 (*PTGS2*), ELAV-like protein 1 (*ELAVL1*), Beclin-1 (*BECN1*), and signal transducer and activator of transcription 3 (*STAT3*) using qPCR, gastrocnemius samples were homogenized in TRIzol, and cultured myotubes were harvested in TRIzol; then, total RNA was isolated from the samples using the Direct-zol RNA Microprep kit (R2062, Zymo Research, Irvine, CA, USA). The concentration and quality of each RNA sample were assessed using the NanoDrop One spectrophotometer (Thermo Scientific, Waltham, MA, USA). RNA integrity was measured with the RNA IQ Assay using a Qubit 4 Fluorometer (Thermo Scientific, Waltham, MA, USA). cDNA was synthesized using an iScript Reverse Transcription Supermix for RT-qPCR (Bio-Rad Laboratories, Hercules, CA, USA, 1708841). PCR reactions used the following primers and were run on a CFX Opus Real-Time PCR System (Bio-Rad Laboratories) with SsoAdvanced Universal SYBR Green Supermix (Bio-Rad Laboratories, Hercules, CA, USA, 1725271): *HMOX1* (qHsaCID0022141), *TFRC* (qHsaCID0022106), *FTH1* (qHsaCED0044830), *NCOA4* (qHsaCED0044830), *SLC40A1* (qHsaCED0005662), *GPX4* (qHsaCID0023890), *SLC7A11* (qHsaCED0045495), ACSL4 (qHsaCED0042270), PTGS2 (qHsaCED0042341), ELAVL1 (qHsaCID0017218), *BECN1* (qHsaCID0016032), *STAT3* (qHsaCID0010912), and the housekeeping gene glyceraldehyde 3-phosphate dehydrogenase (*GAPDH*; qHsaCED0038674) (Bio-Rad Laboratories, Hercules, CA, USA, 10025220). All primers were obtained from and validated by Bio-Rad Laboratories (Bio-Rad Laboratories, Hercules, CA, USA, 10025220). Melt curves were assessed to ensure that only one melt peak was present for each sample. The quantification cycle (Cq) of target genes was normalized to the Cq of the housekeeping gene *GAPDH*, and the delta delta Ct value (ΔΔCt) was determined using the methods outlined by Livak et al. [31]. All samples were run in duplicate, and results were averaged. For statistical analysis, the ΔΔCt was calculated for each sample, and statistical analyses were run on the ΔΔCt values.

### 2.5. Protein Extraction and Western Blot

Muscle samples were homogenized in lysis buffer consisting of 150 mM NaCl, 50 mM Tris-HCl, 1% Triton X-100, 0.5% sodium deoxycholate, 0.1% SDS, and 1x protease inhibitor cocktail (Sigma Aldrich, Burlington, MA, USA, P8340) for protein isolation. Cultured myotubes harvested from 6-well plates were resuspended in the aforementioned lysis buffer and then sonicated with a Bioruptor sonicator (Diagenode SA, Liège, Belgium) for five cycles of 30 s on, 30 s off, at 4 °C. Protein concentration of each sample was determined in duplicate using a commercially available Pierce BCA assay kit (Thermo Scientific, Rockford, IL, USA, 23225) and a Varioskan LUX Multimode microplate reader (Thermo Scientific, Rockford, IL, USA, VL0000D0). Protein samples were mixed with 2x Laemmli sample buffer and 2-mercaptoethanol reducing agent (Bio-Rad Hercules, CA, USA, 1610747). An amount of 20 ug of protein was separated using electrophoresis on 7.5%, 12%, and 4–20% Criterion TGX Precast Midi Protein Gels (Bio-Rad Laboratories, 5671025, 5671045, and 5671095, respectively) in a Criterion Cell Tank (Bio-Rad Laboratories, Hercules, CA, USA). Proteins were transferred to 0.2 µm Amersham Hybond P polyvinylidene difluoride (PVDF) transfer membranes (Cytiva, Malborough, MA, USA, 10600021), and total protein per lane was quantified using Ponceau S staining (Boston BioProducts, Milford, MA, USA, ST180). Membranes were incubated in the primary antibodies overnight, followed by incubation with the appropriate HRP-conjugated secondary antibody. Primary antibodies used were HMOX1 (Invitrogen, Waltham, MA, USA, MA5-31557), GPX4 (Proteintech, Rosemont, IL, USA, 30388-1-AP), SLC7A11 (Proteintech, 26864-1-AP), ACSL4 (Proteintech, 66617-1-Ig), PTGS2 (Proteintech, 66351-1-Ig), ELAVL1 (Proteintech, 66549-1-Ig), Beclin-1 (Proteintech, 11306-1-AP), and 4-Hydroxynonenal (4HNE) (Abcam, Waltham, MA, USA, ab46545). Secondary antibodies used were Goat anti-Rabbit IgG (1:10,000, Invitrogen, Waltham, MA, USA, 31462) or Goat anti-Mouse IgG (1:10,000, Invitrogen, Waltham, MA, USA, 31430). Membranes were visualized using Clarity ECL Substrate (Bio-Rad Laboratories 1705060), and bands were detected using the ChemiDoc MP Imaging System (Bio-Rad Laboratories, Hercules, CA, USA). Band intensities were quantified using Image Lab (Bio-Rad Laboratories, Hercules, CA, USA) and normalized to total protein (Ponceau S staining). Both protein expression and lipid peroxidation data are reported as the mean of each experimental group normalized to the control and displayed as mean ± SD.

### 2.6. Statistical Analysis

Baseline characteristics between PAD patients (IC and CLI) and control subjects were compared using chi-square or Fisher’s exact tests for categorical variables and independent *t*-tests for continuous variables. Significance for group differences in lipid peroxidation and gene or protein expression data from muscle tissue samples was determined by one-way analysis of variance (ANOVA) and evaluated post-hoc by Tukey multiple comparison test. All lipid peroxidation, gene expression, and protein expression data are represented as each group’s relative difference from the control. Significance for group differences in lipid peroxidation and gene or protein expression data from cultured myotubes was determined by an unpaired *t*-test. The assumptions of normality and homogeneity of variances were verified prior to analysis using the Shapiro–Wilk test and Levene’s median test. In the instance that normality was violated, a Kruskal–Wallis test was used, and in the instance that there were unequal variances, a Welch’s adjustment was used. Prism statistical software (version 8.3.0, GraphPad, Boston, MA, USA) was used to perform all statistical analyses. All data are reported as mean ± SD, and significance was accepted at *p* < 0.05.

## 3. Results

### 3.1. Patient Characteristics

Baseline characteristics for all patients and control subjects are reflected in Table 1.

### 3.2. Ferroptosis Gene Expression

#### 3.2.1. PAD Patient Muscle Tissue Gene Expression

**Figure 1:**
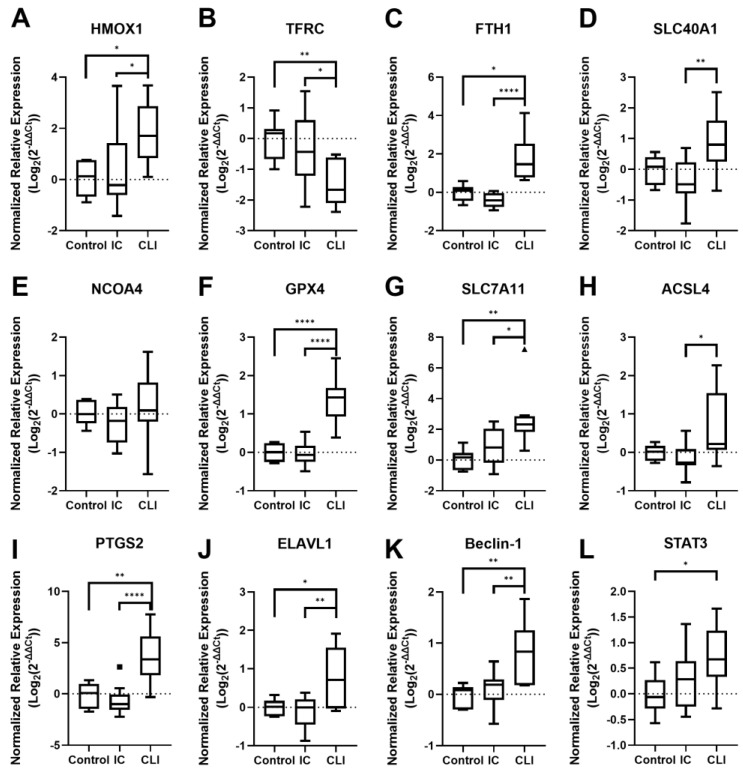
RNA expression of major markers of ferroptosis in the muscle tissue of IC and CLI PAD patients. Graphs show the normalized relative expression of (**A**) *HMOX1*, (**B**) *TFRC*, (**C**) *FTH1*, (**D**) *SLC40A1*, (**E**) *NCOA4*, (**F**) *GPX4*, (**G**) *SLC7A11*, (**H**) *ACSL4*, (**I**) *PTGS2*, (**J**) *ELAVL1*, (**K**) *Beclin-1*, and (**L**) *STAT3* RNA. Control *n* = 7, IC *n* = 10, CLI *n* = 10 for all graphs. * *p* < 0.05, ** *p* < 0.01, **** *p* < 0.0001.

To investigate whether ferroptosis was occurring in the skeletal muscle of both IC and CLI PAD patients, we used qPCR to quantify the expression of ferroptosis-related RNA (Figure 1). The most notable results among the iron metabolism-related genes are the significant increase in *HMOX1* expression as PAD progresses (*p* = 0.0145; Figure 1A) and the corresponding significant decrease in *TFRC* expression (*p* = 0.0095; Figure 1B). Additionally, the expression levels of *FTH1* (*p* = 0.0008; Figure 1C) significantly increased in CLI patients, while *SLC40A1* (Figure 1D) trended upwards in CLI patients (*p* = 0.0911) when compared to controls but only showed a significant difference between IC and CLI patients (*p* = 0.0049). *NCOA4* did not show a strong trend in either direction (Figure 1E). We also examined genes involved in glutathione metabolism, *GPX4* and *SLC7A11*. The expression of both *GPX4* (*p* < 0.0001; Figure 1F) and *SLC7A11* (*p* = 0.0018; Figure 1G) was significantly increased in CLI patients. Similarly, *PTGS2* (*p* < 0.0001; Figure 1I) was significantly upregulated in CLI patients, while *ACSL4* (Figure 1H) showed a strong trend upwards in CLI patients when compared to controls (*p* = 0.0939) but only showed a significant difference between IC and CLI patients (*p* = 0.0154). Additionally, we observed upregulation of autophagy-related genes *ELAVL1* (*p* = 0.0235; Figure 1J) and *BECN1* (*p* = 0.0018; Figure 1K) in CLI patients. The inflammation-related gene *STAT3* was also significantly upregulated (*p* = 0.0268; Figure 1L) in CLI patients. These results suggest that changes in gene expression related to iron metabolism, glutathione metabolism, autophagy, and inflammation may be associated with severe PAD myopathy and potentially indicative of ferroptosis occurring in skeletal muscle tissue.

#### 3.2.2. Myotube Gene Expression Following Ferroptosis Induction with Erastin

**Figure 2:**
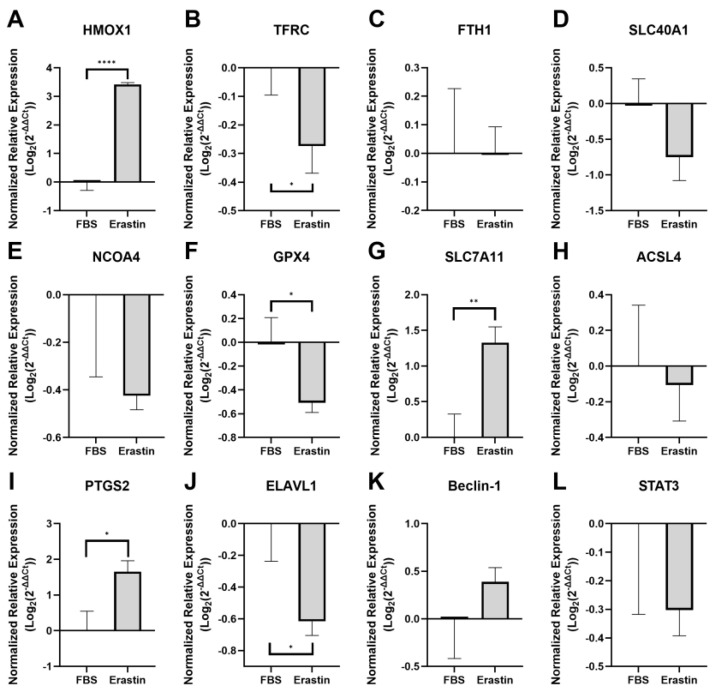
RNA expression of ferroptosis markers in cultured myotubes treated with and without erastin. Graphs show the normalized relative expression of (**A**) *HMOX1*, (**B**) *TFRC*, (**C**) *FTH1*, (**D**) *SLC40A1*, (**E**) *NCOA4*, (**F**) *GPX4*, (**G**) *SLC7A11*, (**H**) *ACSL4*, (**I**) *PTGS2*, (**J**) *ELAVL1*, (**K**) *Beclin-1*, and (**L**) *STAT3* RNA. Control *n* = 7, IC *n* = 10, CLI *n* = 10 for all graphs. * *p* < 0.05, ** *p* < 0.01, **** *p* < 0.0001.

Among the genes related to iron metabolism, the erastin-treated myotubes (erastin myotubes) mimicked the expression patterns of several genes observed in CLI patients. Specifically, *HMOX1* expression significantly increased (*p* < 0.0001; Figure 2A), while *TFRC* expression significantly decreased (*p* = 0.0243; Figure 2B). *FTH1* expression showed no significant change (*p* = 0.9878; Figure 2C), which directly contrasts what was observed in CLI patients. Lastly, although *SLC40A1* (*p* = 0.0523; Figure 2D) and *NCOA4* (*p* = 0.1038; Figure 2E) did not show a significant change, they did exhibit trends of decreased expression, which contradicts what was observed in CLI patients.

In contrast to the PAD patients, *GPX4* expression was significantly decreased in erastin myotubes (*p* = 0.0170; Figure 2F). However, similar to CLI patients, *SLC7A11* was significantly upregulated (*p* = 0.0044; Figure 2G). No significant difference in *ACSL4* (*p* = 0.6692; Figure 2H) expression was observed between FBS-treated myotubes (FBS myotubes) and erastin myotubes, which contrasts with what was observed in CLI patients, while *PTGS2* was significantly upregulated in erastin myotubes (*p* = 0.0104; Figure 2I), matching what was observed in CLI patients.

Comparing the expression of autophagy-related genes, *ELAVL1* was significantly downregulated in erastin myotubes (*p* = 0.0137; Figure 2J), which contradicts our findings in CLI patients, whereas *BECN1* showed a trend of upregulation that was not statistically significant (*p* = 0.2024, Figure 2K). Lastly, the inflammation-related gene *STAT3* exhibited a negative trend in expression in erastin myotubes, though this trend was not statistically significant (*p* = 0.1882; Figure 2L). These findings suggest that there is an overlap in the expression of some ferroptosis-related genes in the erastin-treated myotube model and PAD skeletal muscle tissue; however, there are also key differences.

### 3.3. Protein Expression

#### 3.3.1. PAD Patient Muscle Tissue Protein Expression

**Figure 3:**
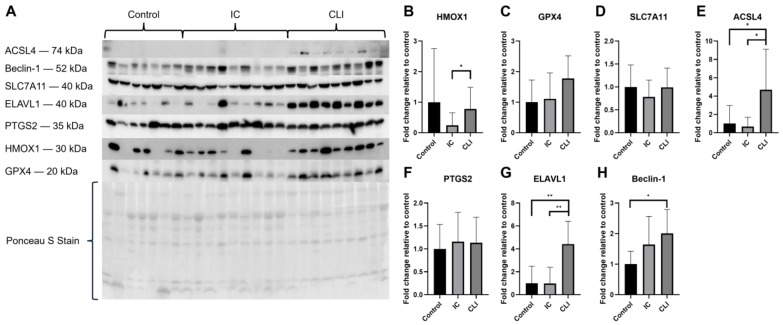
Protein expression in the muscle tissue of IC and CLI PAD patients. (**A**) Western blot results for HMOX1, GPX4, SLC7A11, ACSL4, PTGS2, ELAVL1, and Beclin-1, with a representative Ponceau S stain. Graphs depict the fold change relative to control for (**B**) HMOX1, (**C**) GPX4, (**D**) SLC7A11, (**E**) ACSL4, (**F**) PTGS2, (**G**) ELAVL1, and (**H**) Beclin-1 protein expression. Control *n* = 7, IC *n* = 9, CLI *n* = 9 for all graphs. * *p* < 0.05, ** *p* < 0.01.

To determine if the changes in gene expression translated to changes in protein expression, we quantified several proteins of interest in the muscle tissue of PAD patients. Although HMOX1 did not show a statistically significant change compared to controls (Figure 3B), there was a clear trend of increasing HMOX1 expression in CLI patients, as seen in the Western blot results (Figure 3A). Notably, HMOX1 expression was significantly higher in CLI patients compared to IC patients (*p* = 0.0489). Although GPX4 expression showed a trend towards increasing in CLI patients, statistical significance was not achieved (*p* = 0.1275; Figure 3A,C). SLC7A11 and PTGS2 protein expression did not change significantly (*p* = 0.4863 and *p* > 0.9999; Figure 3D and Figure 3F, respectively) despite increased gene expression in the same samples. Conversely, ACSL4 (*p* = 0.031; Figure 3E), ELAVL1 (*p* = 0.0020; Figure 3G), and Beclin-1 (*p* = 0.0496; Figure 3H) exhibited significantly increased expression in CLI patients. Western blots of PTGS2 and ELAVL1 both displayed bands slightly off from where they were expected, PTGS2 displayed bands around 30–35 kDa, and ELAVL1 displayed bands around 45–50 kDa. It is well-documented that unspecific cleavage products of PTGS2 will display around 39 kDa, so we theorize that these bands are unspecific cleavage products [32]. Additionally, studies have shown that ELAVL1 increases in molecular weight as its concentration increases; the bands observed around 45–50 kDa may be due to either phosphorylation or oligomerization of the ELAVL1 protein [33,34]. Lastly, Western blots were used to quantify lipid peroxidation using an antibody targeting 4HNE, a byproduct of lipid peroxidation and a marker of oxidative stress. In PAD muscle tissue, IC patients showed significantly increased lipid peroxidation (*p* = 0.0330), while CLI patients showed a non-significant trend of increased lipid peroxidation (*p* = 0.2280) (Figure S1A,C).

#### 3.3.2. Erastin-Treated Myotubes Protein Expression

**Figure 4:**
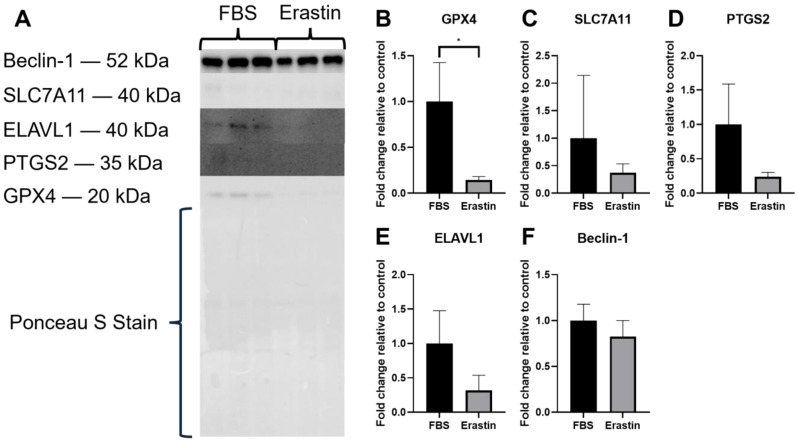
Protein expression in cultured myotubes treated with either FBS or erastin. (**A**) Western blot results for GPX4, SLC7A11, PTGS2, ELAVL1, and Beclin-1, with a representative Ponceau S stain. Graphs depict the fold change relative to control for (**B**) GPX4, (**C**) SLC7A11, (**D**) PTGS2, (**E**) ELAVL1, and (**F**) Beclin-1 protein expression. FBS *n* = 3, erastin *n* = 3 for all graphs. * *p* < 0.05.

Protein extracted from the erastin myotubes was analyzed for the expression of several proteins of interest. Of the 5 proteins quantified, only GPX4 showed a significant change, i.e., was reduced in erastin myotubes (*p* = 0.0254; Figure 4A,B). Of the remaining four proteins, SLC7A11 (*p* = 0.3994; Figure 4C), PTGS2 (*p* = 0.0900; Figure 4D), and ELAVL1 (*p* = 0.0873; Figure 4E) trended towards reduced expression, albeit non-significantly, and Beclin-1 was unchanged (Figure 4F). Lipid peroxidation was elevated in erastin-treated myotubes, but this increase was also not statistically significant (*p* = 0.4019; Figure S1B,D).

## 4. Discussion

The purpose of this study was to investigate the role of ferroptosis in PAD myopathy by quantifying ferroptosis markers across disease stages. By comparing changes in RNA and protein expression patterns between IC and CLI PAD patients, non-PAD control patients, and erastin-treated cultured myotubes, we identified several key pathways that implicate ferroptosis in PAD myopathy, particularly in CLI patients. Our findings suggest that disruptions in iron metabolism and autophagy are potential drivers of ferroptotic cell death in PAD myopathy.

The most compelling evidence for ferroptosis in PAD comes from changes in iron metabolism markers. HMOX1, which generates free iron through heme catabolism, showed increased gene expression in both CLI muscle tissue and erastin-treated myotubes. Although the change in HMOX1 protein abundance did not reach statistical significance, the upward trend noted in the muscle from CLI patients (Figure 3A) paralleled the changes seen in *HMOX1* RNA expression. This increased HMOX1 expression likely contributes to ferroptosis by expanding the labile iron pool (LIP), as previously demonstrated in atherosclerotic smooth muscle cells [35]. Moreover, *FTH1*, involved in the sequestration and storage of excess free iron [36], was also upregulated in CLI muscle, which further supports the intramuscular expansion of iron within our patient samples. Prior studies using endothelial cells also showed that increased ferritin expression serves as a compensatory mechanism against heme-induced iron overload, with ferritin preventing oxidative stress and subsequent tissue injury through its iron sequestration and ferroxidase activity [36]. Thus, the upregulation of *FTH1* RNA observed in CLI PAD patients of the current study could represent a protective mechanism against free iron accumulation, catalyzed by elevated HMOX1.

In contrast, we observed a downregulation of *TFRC*, a cellular iron importer, in both CLI patients and erastin-treated myotubes. These findings were unexpected and contrary to typical ferroptosis models where *TFRC* is upregulated [37]. Nevertheless, reduced *TFRC* expression could also reflect the extent of intramuscular oxidative stress in CLI muscle, as excess reactive oxygen species were previously shown to inhibit the post-translational synthesis of TFRC [38,39]. Although such a reduction in TFRC could be viewed as a protective mechanism against the HMOX1-induced increase in the LIP and subsequent intracellular iron toxicity [38], recent research also suggests TFRC has additional, noncanonical functions beyond iron homeostasis, which include muscle regeneration and the prevention of abnormal lipid accumulation [40,41]. In a mouse model of sarcopenia, researchers found that TFRC ablation in muscle satellite cells impairs muscle regeneration while promoting both labile iron accumulation and lipogenesis [41]. Given that increased intramuscular lipids are a known pathological feature of PAD myopathy and contribute to oxidative stress [42,43,44,45,46], the reduced *TFRC* expression observed specifically in CLI muscle (despite elevated intramuscular *HMOX1* in both IC and CLI patients) could represent an important biomarker of enhanced myopathic disease.

Changes noted in lipid metabolism markers further support the latter hypothesis, as well as the involvement of ferroptosis in PAD myopathy. For instance, ACSL4, which increases cellular sensitivity to ferroptosis by incorporating long-chain polyunsaturated fatty acids (PUFA) into cell membranes, showed significant increases in both gene and protein expression in CLI skeletal muscle [47]. The shift towards higher membrane PUFA content likely sensitizes PAD muscle tissue to oxidative damage and lipid peroxidation. The significant upregulation of PTGS2, widely accepted as a primary ferroptosis biomarker, in both CLI tissue and erastin-treated myotubes provides additional evidence that ferroptotic cell death is occurring in the advanced stages of PAD [48]. Moreover, the intramuscular protein content of the antioxidant markers GPX4 and SLC7A11, key components of the glutathione redox system, was unchanged in CLI patients, despite an increase in gene expression. This upregulation of *GPX4* gene expression, which differs from typical ferroptosis models where *GPX4* is downregulated [49,50], likely represents a compensatory response to increased oxidative stress and lipid peroxidation in CLI patients. However, our findings show that transcriptional upregulation failed to increase protein levels. Thus, the discrepancy between gene and protein expression, particularly evident in CLI patients, indicates that although cells in PAD muscle tissue attempt to enhance their antioxidant defenses through increased transcription, these efforts are ultimately unsuccessful at preventing ferroptosis.

Lastly, a downregulation of TFRC was previously observed in autophagy-deficient cells treated with erastin, suggesting that the relationship between iron metabolism and ferroptosis in PAD may be modulated by autophagy [29,51]. Our analysis of autophagy-related markers also revealed a strong connection between autophagy and ferroptosis in PAD progression. ELAVL1 and BECN1 showed significant increases in both gene and protein expression in CLI skeletal muscle. The increased expression of ELAVL1, which stabilizes *BECN1* RNA, leads to elevated Beclin-1 production and enhanced autophagosome formation [52]. Beclin-1’s dual role in promoting both autophagy and ferroptosis, particularly through its inhibition of system X_c_^−^, via SLC7A11 binding, suggests that increased autophagy may directly contribute to ferroptotic cell death in PAD [53,54]. STAT3 expression was also elevated in our study, though its role appears complex. While STAT3 was previously reported to both inhibit and promote ferroptosis, our findings suggest that any protective effects of STAT3-mediated GPX4 and SLC7A11 upregulation are insufficient to prevent ferroptosis in PAD muscle tissue [55,56].

This study has potential limitations. When our muscle tissue and cell culture findings were compared, the erastin-treated myotubes showed no change in ferritin expression. Past studies have shown that heme uptake may provide a transferrin-independent method of iron import [36,57], and extracellular heme can contribute to various vascular injuries through increased oxidative stress [58,59]. Since CLI PAD patients exhibit significantly elevated serum myoglobin levels [60] and *HMOX1* expression (Figure 1A), this likely provides an alternative route for iron overload in vivo. Given that ferritin and TFRC are both coordinately regulated by iron-regulatory proteins (IRPs) [61], the increase in ferritin and decrease in *TFRC* observed in CLI patients likely result from free iron accumulation in muscle cells due to increased heme uptake and HMOX1-mediated heme degradation. This mechanism explains why *FTH1* expression changed only in CLI muscle tissue and not in erastin-treated myotubes, as the culture medium lacked significant heme sources. However, this pathway does not fully account for the decreased *TFRC* expression observed in cultured myotubes, highlighting the complexity of ferroptosis in PAD and suggesting that ferroptosis in PAD myopathy may involve additional regulatory mechanisms not captured in our simplified cell culture model. Lastly, our study’s reliance on RNA and protein expression patterns, while informative, does not directly measure ferroptotic cell death or iron-dependent lipid peroxidation in situ. Future studies utilizing techniques such as immunohistochemistry to quantify tissue composition for iron deposits would provide additional evidence of ferroptosis in PAD myopathy.

## 5. Conclusions

Despite the noted limitations, our study provides an important foundation for understanding the potential role of ferroptosis in PAD myopathy. The identification of dysregulated ferroptosis-related pathways expands our understanding of the complex pathophysiology underlying PAD-associated muscle degeneration. Our findings suggest that ferroptosis-associated pathways are dysregulated in CLI PAD muscle tissue and may contribute to myopathy. The observed alterations in iron metabolism, lipid peroxidation, and autophagy markers are consistent with ferroptotic processes, though additional research is needed to definitively establish ferroptosis as a causal mechanism in PAD myopathy progression.

Specifically, our analyses identified several dysregulated pathways associated with ferroptosis in CLI muscle tissue, including alterations in iron metabolism (HMOX1), membrane lipid composition (ACSL4), and autophagy regulators (ELAVL1 and Beclin-1). These changes collectively suggest the potential involvement of ferroptotic mechanisms, though the pattern of marker expression differs in some respects from canonical ferroptosis models. The apparent failure of compensatory mechanisms, particularly in the glutathione redox system, suggests that therapeutic strategies targeting iron metabolism, lipid metabolism, or autophagy regulation might be more effective than antioxidant interventions alone. Future research should focus on identifying the upstream regulators of these pathways and whether their modulation could slow or prevent PAD myopathy progression. Additionally, the interplay between extracellular heme, HMOX1, TFRC, and FTH1 in muscle tissue is a particularly exciting mechanism, warranting further exploration. Studies employing specific ferroptosis inhibitors such as ferrostatin-1 or liproxstatin-1 in animal models of PAD would help establish whether targeting ferroptosis can attenuate muscle degeneration and improve functional outcomes, thereby confirming the pathological role of ferroptosis in PAD myopathy.

## Figures and Tables

**Table 1:** Patient Characteristics.

	Control	IC	CLI	*p*-Value
Age, y	69.1 ± 3.4	67.1 ± 8.1	63.3 ± 5.6	0.208
Sex, No. (%)				**0.** **018**
Male	7 (100)	10 (100)	6 (60)	
Race, No. (%)				**<0.001**
White	7 (100)	10 (100)	1 (10)	
Black	0 (0)	0 (0)	3 (30)	
Hispanic	0 (0)	0 (0)	6 (60)	
Comorbidities, No. (%)				
Coronary Artery Disease	0 (0)	3 (30)	3 (30)	0.259
Hypertension	7 (100)	7 (70)	6 (60)	0.168
Dyslipidemia	5 (71)	8 (80)	6 (60)	0.617
Diabetes	1 (14)	2 (20)	9 (90)	**0.0** **01**
Smoking status, No. (%)				**<0.001**
Never smoked	3 (43)	0 (0)	8 (80)	
Current smokers	1 (14)	8 (80)	1 (10)	
Former smokers	3 (43)	2 (20)	1 (10)	
ABI	1.10 ± 0.18	0.69 ± 0.26 *	0.18 ± 0.21 *^,#^	**<0.001**

Note: *p*-value corresponds to the main effect, with a significant main effect highlighted in bold, * = significantly different from Control, ^#^ = significantly different from IC.

## Data Availability

The data that support the findings of this study are available from the corresponding author upon reasonable request.

## Supplementary Materials

The following supporting information can be downloaded at: https://www.mdpi.com/article/10.3390/biology14050537/s1, Figure S1: Quantified Lipid Peroxidation Data and 4HNE Western Blot Results. (A) Relative Lipid Peroxidation levels in IC and CLI patients compared to controls. (B) Relative lipid peroxidation levels in erastin-treated myotubes compared to FBS-treated myotubes. (C) Western blot of 4HNE expression of Control, IC, and CLI patients, with Ponceau S stain. (D) Western blot of 4HNE expression in FBS-treated myotubes and erastin-treated myotubes with Ponceau S stain. Control *n* = 7, IC *n* = 9, and CLI *n* = 9 for (A) and (C). FBS *n* = 3 and erastin *n* = 3 for Figures (B) and (D). * *p* < 0.05.

## Abbreviations

The following abbreviations are used in this manuscript:

PAD	Peripheral artery disease
IC	Intermittent claudication
CLI	Critical limb ischemia
IRI	Ischemia/reperfusion injury
HMOX1	Heme oxygenase 1
TFRC	Transferrin receptor 1
FTH1	Ferritin heavy chain 1
NCOA4	Nuclear receptor coactivator 4
FPN	Ferroportin
SLC40A1	Solute carrier family 40 member 1
GPX4	Glutathione peroxidase 4
SLC7A11	Solute carrier family 7 member 11
ACSL4	Acyl-CoA synthetase long-chain family member 4
PTGS2	Prostaglandin-endoperoxide synthase 2
ELAVL1	ELAV-like protein 1
STAT3	Signal transducer and activator of transcription 3
ABI	Ankle brachial index
4HNE	4-Hydroxynonenal
LIP	Labile iron pool
NRF2	Nuclear factor erythroid 2-related factor 2
