# Supplementary material for: Deciphering the Role of Ferroptosis in the Pathogenesis of Peripheral Artery Disease Myopathy
Source: Biology (Basel). 2025 May 12;14(5):537. doi: 10.3390/biology14050537 (PMC12108827; doi:10.3390/biology14050537)
Supplement: Supplementary file 1 [file biology-14-00537-s001.zip › biology-3567499-supplementary.pdf]

biology

MDPI

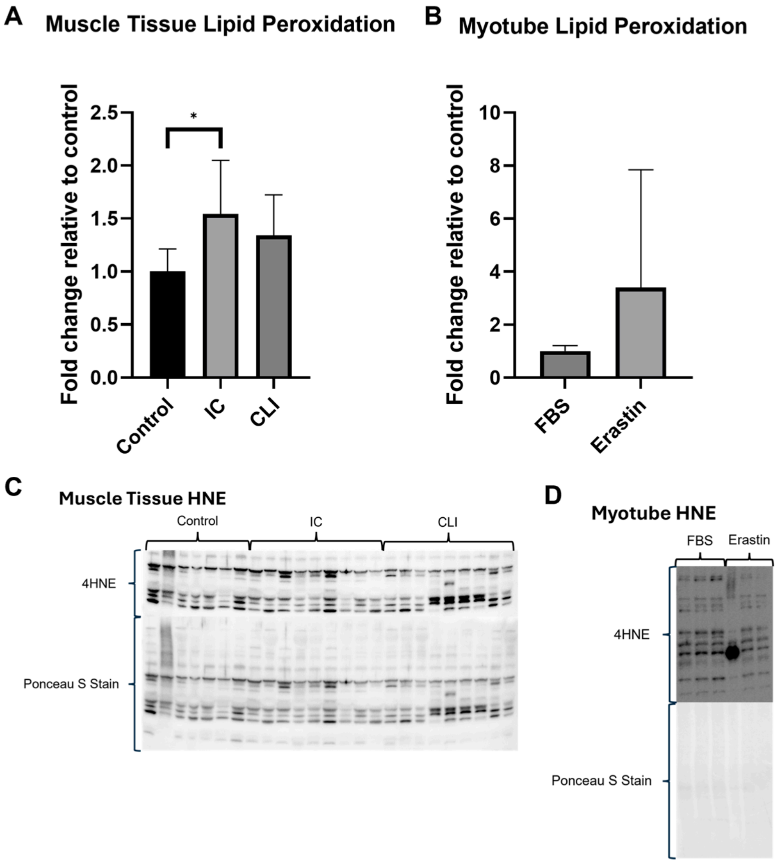

Supplementary Figure S1. Quantified Lipid Peroxidation Data and 4HNE Western Blot Results. (A) Relative Lipid Peroxidation levels in IC and CLI patients compared to controls. (B) Relative lipid peroxidation levels in erastin-treated myotubes compared to FBS-treated myotubes. (C) Western blot of 4HNE expression of Control, IC and CLI patients, with Ponceau S stain (D) Western blot of 4HNE expression in FBS-treated myotubes and erastin-treated myotubes with Ponceau S stain.
Control $n = 7$, IC $n = 9$, and CLI $n = 9$ for (A) and (C). FBS $n = 3$ and erastin $n = 3$ for Figure (B) and (D).
* $p < 0.05$.
